# Web-based real-time risk assessment of coronavirus disease 2019 infection in schools and social dining settings

**DOI:** 10.1016/j.nmni.2025.101600

**Published:** 2025-05-20

**Authors:** Yuta Okada, Minami Ueda, Hiroshi Nishiura

**Affiliations:** Graduate School of Medicine, Kyoto University, Kyoto, 606-8503, Japan

**Keywords:** COVID-19, Indoor transmission, Aerosol, Web application, Japan

## Abstract

**Background:**

Schools and dining situations are associated with a high risk of indoor transmission of coronavirus disease 2019 (COVID-19). Performing risk assessment in real time could enable organizers to adjust the duration and size of indoor activities depending on the epidemic situation.

**Methods:**

The per-hour transmission rates of COVID-19 from a single infector in school and social dining settings were estimated from COVID-19 surveillance data in Japan from January to February 2022 using a mathematical model. We then developed a web application that calculates the risk of COVID-19 infection in those settings, accounting for place of residence, vaccination history, duration of indoor activity, and the number of participants.

**Results:**

The estimated per-hour transmission rates were 0.01934 (95 %CrI: 0.01939–0.01947) in social dining settings and 0.00324 (95 %CrI: 0.00323–0.00325) in school settings. Accounting for the epidemiological risk of having infected persons in indoor settings, a web application was devised to compute the risk of a single participant contracting COVID-19 at the event. Web application users can vary input variables including the duration and the number of participants, thereby benefiting the real time risk reduction.

**Conclusions:**

The per-hour transmission rate was higher in social dining settings compared with that in school settings, but the greater number of participants that is typical of gatherings at schools might offset this gap in per-hour per-infector transmission risk. The proposed web application can act as an important tool for promoting risk awareness regarding COVID-19 in high-risk settings.

## Highlights

• We quantified the per-hour risk of coronavirus disease 2019 by social setting.
• Transmission rates in schools and social dining were estimated.
• A web application was developed to visualize the risk of indoor transmission.
• Depending on the epidemic situation, the duration and size can be decided.

## 1 Introduction

One of the key public health issues during the coronavirus disease 2019 (COVID-19) pandemic was determining how to control COVID-19 transmission effectively. In this context, various epidemiological studies were performed to identify the locations or specific societal settings associated with a high risk of COVID-19 transmission. In general, indoor gatherings with longer duration and a greater number of participants are considered to be hotspots of COVID-19 transmission, and differences in transmission risk by the type of gathering were reported in epidemiological studies on transmission cluster occurrence [[1], [2], [3], [4], [5]]. As the fundamental mechanism of transmission in indoor settings, evidence indicates that closer contact results in increased transmission via droplets, and a longer duration of contact leads to a substantial increase in transmission via aerosols, although the role played by these distinct routes of transmission differs by the extent of overcrowding, mask use, the type of indoor activity, and ventilation rate [[6], [7], [8], [9], [10], [11]].

Despite these epidemiological insights, a substantial proportion of the population does not currently take effective preventive measures such as mask wearing in high-risk indoor settings or frequent indoor ventilation [1,[12], [13], [14]]. In addition to the lack (or cancellation) of governmental mandates for universal masking, two key factors may contribute to this decreased adherence to preventive measures against COVID-19. First, “pandemic fatigue” involves a decrease in adherence to required mitigation measures against COVID-19, including avoidance of social gatherings or mask wearing [[15], [16], [17]]. Second, risk awareness toward the local or global epidemic situation diminished following the downgraded public health risk status of COVID-19. Most countries, including Japan, no longer maintain detailed epidemiological surveillance led by national or local public entities, and media coverage of surveillance is greatly reduced compared with the pre-vaccination period from 2020 to 2021. Additionally, it has become challenging for the general public to understand the disease burden of COVID-19 from direct and indirect deaths, and post-acute sequelae [[18], [19], [20]].

In Japan, the expert committee on COVID-19 proposed the “3Cs” concept (closed spaces, crowded places, and close contact settings) that highlighted high-risk transmission settings, especially in indoor environments on the basis of surveillance and insight into transmission cluster occurrence [21]. The “3Cs” concept was widely adopted by the general public in Japan, and helped promote risk awareness in the early pandemic phase. However, now that the risk awareness has greatly decreased over time, different approaches for promoting risk recognition must be considered. One feasible approach is quantifying and visualizing the risk of contracting COVID-19 in an accessible and real time manner. Several web applications have been developed to display the risk of COVID-19 by events or settings [[22], [23], [24], [25]]. However, approaches that visualize the risk of COVID-19 in high-risk gatherings on the basis of insight from epidemiological data in Japan have not been reported. To fulfill this need, the first step is estimating the per-hour transmission rate of COVID-19 in high-risk settings. Once the transmission rate in a specific space is estimated, the probability of infection via the gathering of interest is calculable on the basis of information such as the setting and duration of the gathering, number of participants, and population-level incidence rate.

The present study aimed to estimate the per-hour transmission rates in school and social dining settings. On the basis of these estimated transmission rates, we developed a web application to perform risk assessment of COVID-19 in a specific indoor setting in real time.

## 2 Materials and methods

### 2.1 Epidemiological data

The present study used data from cases that were confirmed using reverse transcription polymerase chain reaction (RT-PCR) or lateral flow rapid antigen tests, and the daily count of cases stratified by age group was extracted from the electronic surveillance system, referred to as the Health Center Real-Time Information-sharing System on COVID-19 (HER-SYS). For the estimation of the population-level immunity against symptomatic COVID-19 infection, we used national vaccination records in Japan, retrieved from the Vaccination Record System (VRS). This record includes information about the daily number of vaccinations, classified by 5-year age groups. In the subsequent analysis relevant to the current situation with Omicron variant epidemics, we focused on data from January to February 2022. This was because of three reasons: 1, the Omicron variant was prevalent nationwide (including Hiroshima prefecture), 2, the dataset on COVID-19 transmission routes in Hiroshima prefecture (described below) was referable, and 3, the population-level immunity was estimable (based on the method described in the following section), because of the relatively simple immune profile up to the early phase of the Omicron era with small fractions of those experiencing reinfections or having hybrid immunity by both vaccine and infection. (See Supplementary Information for details on the epidemic situation in Hiroshima during this period).

In the present study, we specifically focused on school settings and dining situations, because the risk of clustering in these settings was found to be greater than that in other settings in our earlier study [5]. The dataset regarding the proportion of COVID-19 cases that were attributed to specific transmission settings via contact tracing in Hiroshima prefecture was retrieved [26]. Data were available from January to February 2022, suggesting that social dining was responsible for at least more than 30 % of confirmed cases as of the report on Jan 6, when no regionwide countermeasures (including lock-downs) focusing on eateries and bars were carried out in Hiroshima prefecture as part of the so-called “priority measures to prevent the spread of COVID-19”. (descriptions of the countermeasure are provided in the Supplementary Information) In approximately 10 % of cases, COVID-19 was considered to have been acquired in schools (i.e., primary, junior high, high schools, and universities), whereas no responsible route of transmission was identified for around 40–60 % of cases during this period [27]. We assumed that the attributable proportions of cases in social dining and schools nationwide in Japan mirrored those of Hiroshima.

Other than COVID-19-related data, population statistics as of October 1st, 2023 were used in the present study and retrieved from the Statistics Bureau, Ministry of Internal Affairs and Communications [28].

### 2.2 Estimation of population-level immunity

We estimated the population-level immunity against symptomatic Omicron BA. 1/2 infection for decadal age groups 0–9, 10–19, …, 80 and older, following the methodology described previously by Sasanami et al. [29] For simplicity, we used the median of estimated number of immune people in each age group. The susceptible population in age group a on day t, $S_{t}^{a}$, was derived by subtracting the number of those immune from the total age-specific population.

### 2.3 Infection-age dependent infectivity of severe acute respiratory syndrome coronavirus 2 (SARS-CoV-2)

The proportion of infectious COVID-19 cases as a function of time since infection (i.e., infection age) was modeled on the basis of two previous studies [30,31]. We assumed that the PCR positivity curve by Hellewell et al. with a cycle threshold of 28 corresponded to the proportion of infectious individuals maintaining infectiousness. To adjust this positivity curve to be relevant for Omicron BA.1 or Omicron BA.2, we referred to Russell et al. [31] for the peak of the cycle threshold and the duration of viral load detectability by PCR. For simplicity, we assumed ${g_{\tau }}^{+}$, the probability of PCR positivity over time since infection, as follows:

$${g_{\tau }}^{+}=\left\{ \begin{array}{c} 0.13\tau ,if\;\tau =0,1,\ldots ,5\\ 0.65-0.05\left(\tau -5\right),if\;\tau =6,7,\ldots ,18 \end{array}\right.\text{.}$$

### 2.4 Estimation of the per-hour transmission rate of COVID-19

Let *L* represent major location categories (i.e., school or social dining) and let *l* represent their subcategories as described in the supplementary materials and in Ueda et al. [5] Then, we modeled $\mu_{L,t}$, the expected number of new infections by date of infection in an event at location category L, as a summation of the expected number of infections over location subcategory l in L:

$$\mu_{L,t}=\beta_{L}\sum_{l\in L}\tau_{l}s_{l,t}J_{l,t}\text{.}$$

Here, $\beta_{L}$ is the per-hour transmission rate per single infector, $\tau_{l}$ is the mean duration of time spent in subcategory location *l*, and $s_{l,t}$ and $J_{l,t}$ represent the susceptible proportion and the number of infectious persons in the subcategory location l, respectively, described as follows:

$$s_{l,t}=\sum_{a}\pi_{l}^{a}\frac{S_{t}^{a}}{N^{a}}\text{,}$$

$$J_{l,t}=\sum_{a}n_{l}\pi_{l}^{a}\sum_{\tau =0}^{\infty }{g_{\tau }}^{+}\circ \frac{I_{t-\tau }^{a}}{N^{a}}\text{,}$$

where $\pi_{l}^{a}$ is the age distribution among $n_{l}$, and ${g_{\tau }}^{+}$ is the probability of infectious persons on day τ since date of infection. $S_{t}^{a}$ is the estimated number of susceptible individuals (estimated as described above), $I_{t}^{a}$ is the estimated number of infections by date of infection, and $N^{a}$ is the population size of age group a at the national level. The derivation of $n_{l}$ and $\pi_{l}^{a}$ were based on the population statistics as well as event frequency and user information in Japan that was collected elsewhere [5,32]. (See Supplementary Methods for detailed assumptions and derivations).

We estimated $\beta_{L}$ for schools and social dining by fitting the following model to the case count data from Jan 20 to Feb 28, 2022, when the Omicron (B.1.1.529) subvariants BA.1 and BA.2 were dominant (nationwide, including Hiroshima) and data on suspected transmission settings from Hiroshima prefecture were available:

$$Y_{L,t}∼Poisson\left(\lambda_{L,t}\right)\text{,}$$

$$\lambda_{L,t}=\sum_{\tau =0}^{t}g_{\tau }^{lag}\mu_{L,t-\tau }\text{,}$$

t=Jan20−Feb28,2022,

where $Y_{L,t}$ is the number of cases attributable to transmission in either schools or social dining on day t, and $\lambda_{L,t}$ is the expected number of infections by date of reporting, and $g_{\tau }^{lag}$ is the delay from infection to reporting (see Supplementary Methods for derivation). We set $Y_{L,t}$ for social dining and schools at 30 % and 10 % of total cases from Jan 20 to Feb 28, 2022, respectively, throughout the period, on the basis of data from Hiroshima and by assuming that a substantial portion of cases without known transmission routes was actually exposed in social dining settings [26].

$\beta_{L}$ for schools or social dining was estimated by Bayesian inference using the Markov Chain Monte Carlo (MCMC) method, with weakly informative priors (see Supplementary Methods). We generated 1500 MCMC samples from four chains, discarding 500 warm-up iterations from each chain. Convergence was confirmed by an R-hat value below 1.01, and the confirmation that no divergent chains were observed.

### 2.5 Modeling of the infection probability per hour by different settings

The probability of a person i becoming infected at a single gathering at location category $L_{i}$ can be described as

$$p_{i}=1-e^{-S_{i}\Lambda_{i}}\text{,}$$

where $\Lambda_{i}$ is the hazard of infection by infectors (other than person i) in the same gathering, and $S_{i}$ is the susceptibility of person i. The hazard $\Lambda_{i}$ can be decomposed as:

$$\Lambda_{i}=\beta_{L_{i}}\tau_{L_{i}}J_{L_{i},t}^{{pref}_{i}}\text{,}$$

where $\beta_{L_{i}}$ is the per-hour transmission rate, $\tau_{L_{i}}$ is the duration (hours), and $J_{L_{i},t}^{{pref}_{i}}$ is the expected number of infectors joining the same gathering, which changes depending on the epidemic situation in prefecture $pref_{i}$. We derived $J_{L_{i},t}^{{pref}_{i}}$ assuming that the fraction of infectors $J_{L_{i},t}^{{pref}_{i}}$ among the number of participants $n_{L_{i}}$ is proportional to the prefectural prevalence:

$$J_{L_{i},t}^{{pref}_{i}}=n_{L_{i}}\frac{I_{t}^{pref_{i}}}{N^{pref_{i}}}=n_{L_{i}}\frac{\sum_{a}\sum_{\tau =0}^{\infty }g_{\tau }\circ I_{t-\tau }^{a}}{N^{pref_{i}}}\text{,}$$

where $n_{L_{i}}$ is the number of participants (excluding person i), $I_{t}^{pref_{i}}$ and $N^{pref_{i}}$ are the total infector and population in the prefecture of interest, respectively.

Because the daily COVID-19 incidence by age at the national and the prefectural level in Japan from universal surveillance after May 8th, 2023 was not available, we used the following approximation for $J_{l_{i},t}$:

$$J_{L_{i},t}\approx n_{L_{i}}\frac{I_{pref_{i},w_{t}}\frac{\sum_{\tau =0}^{\infty }g_{\tau }}{7}}{N^{pref_{i}}}\approx n_{L_{i}}\frac{I_{pref_{i},w_{t}}\times 0.836}{N^{pref_{i}}}\text{,}$$

where $I_{pref_{i},w_{t}}$ is the total estimated number of COVID-19 cases at the prefectural level in the week of interest, as derived from our previous study. Note that $I_{pref_{i},w_{t}}$ was only available for 33 out of 47 prefectures in Japan, and the data for Sep 13–20, 2023 were used [33].

### 2.6 Visualization of infection risk from a single gathering

We built a web application to visualize the probability of COVID-19 on the basis of the input variables by users. The probability of a person i being infected after attending the same gathering k times can be calculated as:

$$p_{i}^{\left\{k\right\}}=1-e^{-kS_{i}\Lambda_{i}},k=1,2,\ldots \;\text{.}$$

The susceptibility $S_{i}$ is designed to reflect the user's answer to “Have you had COVID-19 or been vaccinated in the last 6 months?” For simplicity, we defined $S_{i}$ as follows:

$$S_{i}=\left\{ \begin{array}{c} 0.5,if\;answer{=}^{\prime \prime }Yes^{\prime \prime }\\ 1.0,if\;answer{=}^{\prime \prime }No^{\prime \prime } \end{array}\right.\text{.}$$

On the basis of the user's input for $L_{i}$, $\tau_{i}$, $n_{L_{i}}$, ${pref}_{i}$, and $S_{i}$, we visualized $p_{i}^{\left\{k\right\}}$ (k=1,2,3,4,5) in a web application (English version: https://idd-analysis.shinyapps.io/prob_infect_en/, Japanese version: https://idd-analysis.shinyapps.io/prob_infect_jp/).

### 2.7 Software

All statistical and numerical analyses were performed using R version 4.2.2 [34] and CmdStan version 2.33.0 [35]. The web application was implemented using the Shiny R application [36].

## 3 Results

**Fig. 1:**
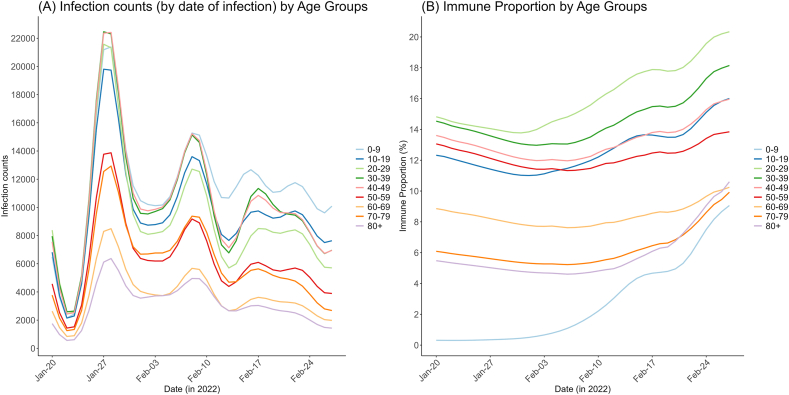
Model of weekly estimates of universal and sentinel COVID-19 counts beyond May 8, 2023 in Tokyo, Osaka, Nara, and Okinawa. Fig. 1: Infection counts and immune proportion by age group at the national level. Panel (A) shows the COVID-19 infection counts in 0–9, 10–19, …, 80+ age groups by date of infection during the study period, which was estimated by back-calculation of the case counts by date of reporting using the delay distributions from infection to reporting. Panel (B) shows the estimated fraction of the population that have protection against symptomatic COVID-19 infection in 0–9, 10–19, …, 80+ age groups.

Fig. 1 shows (A) the infection count (by the date of infection) and (B) the estimated immune proportion by age groups. The immune proportion is especially low among younger age groups because of their low vaccination coverage (see Fig. 1(B)).

Table 1 shows the estimated values of $\beta_{L}$. The value for social dining setting per hour was about six times higher than that for school settings. We used the median values of $\beta_{L}$ for subsequent use in the web application.

**Table 1:** Estimated values of $\beta_{L}$, the per-hour transmission rate of COVID-19 at school and a social dining setting. In the right column, the median and 95 % credible interval (CrI) are shown for each setting (in the left column).

Location type	Estimated Median (95 % CrI)
Schools	0.00324 (0.00323–0.00325)
Social dining	0.01934 (0.01939–0.01947)

∗Numbers in brackets are upper and lower bounds of 95 % credible intervals.

**Fig. 2:**
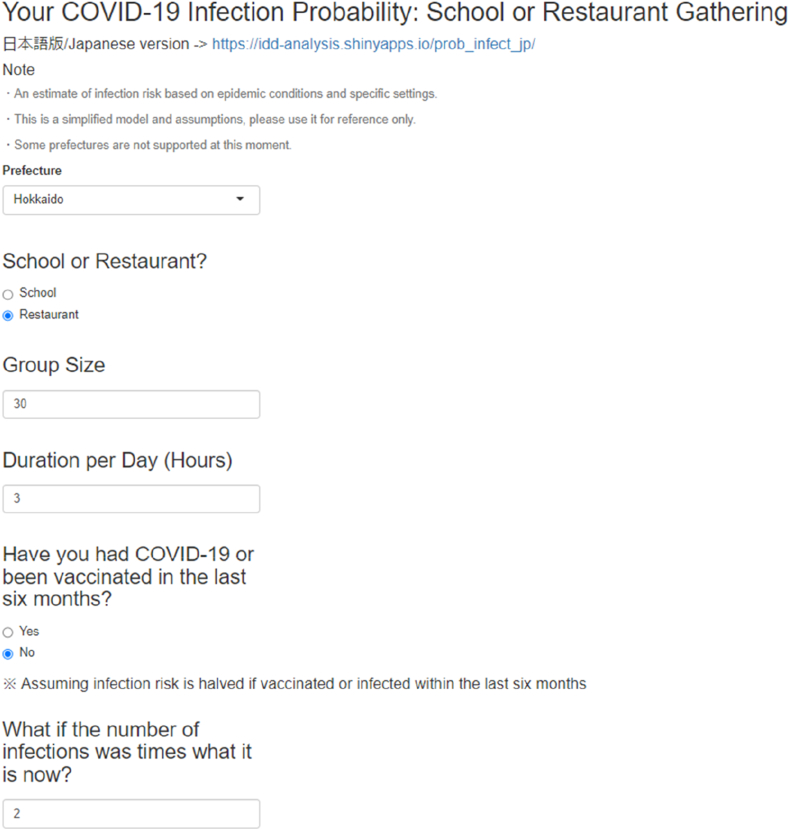
Visual appearance of the web application's input sections for users. Users are expected to give inputs such as prefectures, type of location, number of participants, duration of the gathering, recent exposure to COVID-19 or vaccination. The user can also adjust the multiplier for the infection hazard that is used in the counterfactual scenario.

The web application was built as follows. First, we created the input section, in which users give inputs that are reflected in the subsequent calculation. The visual appearance of the input sections for users of the web application is shown in Fig. 2. Users are expected to provide inputs in the following subsections:

1. Prefecture (i.e., location of residence)
2. The type of setting (“School” or “Restaurant”)
3. Duration of use (Hours)
4. History of vaccination or infection in the last 6 months
5. Multiplier for actual incidence rate used in counterfactual scenarios

**Fig. 3:**
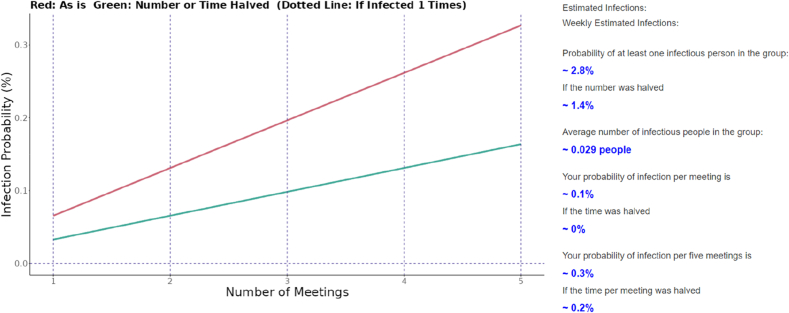
Visual appearance of the web application's output section. On the basis of user inputs such as prefecture, type of location, number of participants, duration of the gathering, recent exposure to COVID-19 or vaccination, the probability of infection per gathering is calculated. The probability of infection when the same gathering is held 1–5 times is shown in the plot on the left (red line), together with a green line that represents the probability of infection in the “what if the number of users or the duration of the gathering was halved?” scenario. On the right side of the plot, supplementary results including the expected number of infections in the gathering are shown as outputs.

Fig. 3 shows an example of the results shown in the web application for Tokyo prefecture. On the right, a list of outputs is displayed:

- probability of infection by the number of meetings
- probability of having at least one infectious person among participants
- probability of having at least one infectious person in the group
- average number of infectious people in the group
- user's probability of infection per meeting/per five meetings
- probability of infection when the time per meeting was halved

With the plot showing the probability of infection together and other outcomes as listed on the right of the plot, these results provided various quantitative results for users to grasp the risk of infection at the gathering of interest.

**Fig. 4:**
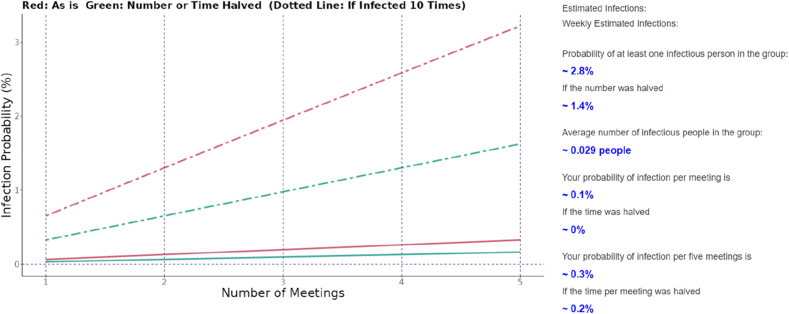
Visual appearance of the web application's output section with results from counterfactual incidence rate. Visual appearance of the web application's output section with the same input as that in Fig. 3, but with additional results on the plot representing the “what if the population-level incidence rate was 10 times higher?” counterfactual scenario.

Fig. 4 shows the same results shown in Fig. 3, except that the counterfactual dotted lines that represent the infection probability in the scenario “what if population-level incidence rate was 10 times the actual incidence rate” are added. The multiplier, arbitrarily set at 10 in Fig. 4, can be manually varied by the user in the range of 0.01–100.

## 4 Discussion

The present study quantified the per-hour transmission rate of COVID-19 from an infected individual at school and in a social dining setting. On the basis of these results, we built a user-friendly web application that allows users to quantitatively understand the risk of contracting COVID-19 that varied not only as a function of the local prevalence (i.e., local epidemic situation) but also by various factors including the duration and the number of participants. Using the web application, organizers could judge the absolute risk of infection and make decisions about plans for future gatherings (e.g., the duration and number of people occupying an indoor space).

Importantly, the current findings indicate that the per-hour transmission rate can be estimated for different social settings and the estimate can be used to calculate the individual-level risk of COVID-19 associated with a gathering. Our modeling of location-specific infection hazard as a function of time was similar to the method used by Tupper et al. to model the reproduction number per event [37], although we focused on estimating the nationwide average transmission rate per hour. Regarding our approach to estimating the transmission rates by settings of interest, explicit estimation of transmission rates solely from population-level data without further contact tracing information has not been done elsewhere, to the best of our knowledge. About our results, the estimated values of transmission rate in our study suggest a higher risk of per-infector infection in social dining settings compared with that in a school setting, in accord with previous studies on superspreading events and transmission clusters [5,[38], [39], [40]]. However, it should also be noted that the level of infection risk in schools may be comparable to that of social dining, given that gatherings at schools typically involve more participants than gatherings in social dining settings.

Another important aspect of the present study was the development of a user-friendly web application that visualizes the probability of infection on the basis of user inputs. Given that the number of people who take precautions such as mask-wearing or indoor ventilation is decreasing, promoting risk awareness against COVID-19 is important not only for the promotion of individual health but also for COVID-19 mitigation at the societal level. Although a similar approach for promoting risk awareness in mass-gathering settings has been reported elsewhere [22], our web application is original in that it is based on the per-hour transmission rates estimated from epidemiological data in Japan. This is distinct from other approaches that are more reliant on theoretical models for aerosol-mediated airborne transmission, and differs from a previously developed web application in Japan that focused on the probability of an infectious person being present in a mass-gathering of interest [7,22,24,25,41].

The current study involved several limitations that should be considered. First, we posed several assumptions when estimating per-hour transmission rates. The fraction of total cases that were attributable to schools or social dining settings was fixed in our study, and the age distribution of the individuals at each type of location was set arbitrarily. Additionally, we assumed the same transmission rates for all locations, which did not account for local variation in the risk of infection, which may have affected the validity of our results. Regarding school settings, published studies from Japan report transmission rates suggest the overall transmission probability of around 1–4 % among contacts to infected cases, which are comparable to our estimates [42,43]; however, to the best of our knowledge, no literature provide any support or validation for our estimates regarding social dining settings. Further improvement of our framework is warranted when large amounts of epidemiological data from finer-scale epidemiological surveys at high-risk settings become available, though potential privacy issues regarding personal-level data especially from mobile devices should be overcome. Neglecting the changes and variations in the adherence to preventive measures including mask-wearing, social distancing, or factors such as the room size or ventilation are further limitations of the present study. Given that there are both population-level or setting-specific real-world evidence on the effectiveness of masks [[44], [45], [46]], social distancing [47,48], or ventilation [46,49], a natural extension of this study will be to specifically estimate the effects of these factors in the setting inside Japan. Once additional data are available to estimate the effects of these factors on transmission rates, we may incorporate these effects into our web application.

In conclusion, we estimated the per-hour, per-infector probability of COVID-19 transmission using a mathematical model and built a web application to visualize the probability of COVID-19 in schools and social dining settings. Our web application enables users to understand the change of infection probability depending on user inputs, and thus may promote both the risk awareness and risk preparedness of its users.

## CRediT authorship contribution statement

**Yuta Okada:** Writing – review & editing, Writing – original draft, Visualization, Methodology, Investigation, Data curation, Conceptualization. **Minami Ueda:** Writing – review & editing, Methodology, Investigation, Conceptualization. **Hiroshi Nishiura:** Writing – review & editing, Writing – original draft, Validation, Supervision, Methodology, Investigation, Conceptualization.

## Ethical approval statement

Ethical approval was not required since all data used in the present study did not include any personally identifiable information.

## Data availability

We were granted access to data from HER-SYS exclusively for epidemiological analysis of COVID-19. Therefore, data from HER-SYS in the present study is not disclosable. Other than data from HER-SYS, the data used in this study are publicly available data from the websites of the Ministry of Health, Labour and Welfare, prefectural governments, and the Ministry of Internal Affairs and Communications. None of the data used in the present study contained personally identifiable information.

## Funding sources

This study was financially supported by the Daikin GAP Fund of Kyoto University. Y.O. received funding from the SECOM Science and Technology Foundation. H.N. received funding from Health and Labour Sciences Research Grants (grant numbers 20CA2024, 21HB1002, 21HA2016, and 23HA2005), the Japan Agency for Medical Research and Development (grant numbers JP23fk0108612 and JP23fk0108685), JSPS KAKENHI (grant numbers 21H03198 and 22K19670), the Environment Research and Technology Development Fund (grant number JPMEERF20S11804) of the Environmental Restoration and Conservation Agency of Japan, Japan Science and Technology Agency SICORP program (grant numbers JPMJSC20U3 and JPMJSC2105), the CREST program (grant number JPMJCR24Q3), and RISTEX program for Science, Technology, and Innovation Policy (grant number JPMJRS22B4). The funders had no role in the study design, data collection and analysis, decision to publish, or preparation of the manuscript.

## Declaration of competing interest

The authors declare that they have no known competing financial interests or personal relationships that could have appeared to influence the work reported in this paper.

## References

[1] Abeya S.G., Barkesa S.B., Sadi C.G., Gemeda D.D., Muleta F.Y., Tolera A.F. (2021). Adherence to COVID-19 preventive measures and associated factors in Oromia regional state of Ethiopia. PLoS One.

[2] Nishiura H., Oshitani H., Kobayashi T., Saito T., Sunagawa T., Matsui T. (2020). Closed environments facilitate secondary transmission of coronavirus disease 2019 (COVID-19). medRxiv.

[3] Pauser J., Schwarz C., Morgan J., Jantsch J., Brem M. (2021). SARS-CoV-2 transmission during an indoor professional sporting event. Sci Rep.

[4] Qian H., Miao T., Liu L., Zheng X., Luo D., Li Y. (2021). Indoor transmission of SARS-CoV-2. Indoor Air.

[5] Ueda M., Hayashi K., Nishiura H. (2023). Identifying high-risk events for COVID-19 transmission: estimating the risk of clustering using nationwide data. Viruses.

[6] Azimi P., Keshavarz Z., Cedeno Laurent JG., Stephens B., Allen J.G. (2021). Mechanistic transmission modeling of COVID-19 on the Diamond Princess cruise ship demonstrates the importance of aerosol transmission. Proc Natl Acad Sci U S A.

[7] Balkan B.A., Chang Y., Sparnaaij M., Wouda B., Boschma D., Liu Y. (2024). The multi-dimensional challenges of controlling respiratory virus transmission in indoor spaces: insights from the linkage of a microscopic pedestrian simulation and SARS-CoV-2 transmission model. PLoS Comput Biol.

[8] Bazant M.Z., Bush J.W.M. (2021). A guideline to limit indoor airborne transmission of COVID-19. Proc Natl Acad Sci U S A.

[9] Dixit A.K., Espinoza B., Qiu Z., Vullikanti A., Marathe M.V. (2023). Airborne disease transmission during indoor gatherings over multiple time scales: modeling framework and policy implications. Proc Natl Acad Sci U S A.

[10] Haddrell A., Oswin H., Otero-Fernandez M., Robinson J.F., Cogan T., Alexander R. (2024). Ambient carbon dioxide concentration correlates with SARS-CoV-2 aerostability and infection risk. Nat Commun.

[11] Shen Y., Li C., Dong H., Wang Z., Martinez L., Sun Z. (2020). Community outbreak investigation of SARS-CoV-2 transmission among bus riders in eastern China. JAMA Intern Med.

[12] Butty A., Bühler N., Pasquier J., Dupraz J., Faivre V., Estoppey S. (2022). Adherence to coronavirus disease 2019 preventive measures in a representative sample of the population of the canton of vaud, Switzerland. Int J Publ Health.

[13] Kusama T., Kiuchi S., Takeuchi K., Ikeda T., Nakazawa N., Kinugawa A. (2022). Information usage and compliance with preventive behaviors for COVID‐19: a longitudinal study with data from the jacsis 2020/JASTIS 2021. Health Care.

[14] Varas S., Elorrieta F., Vargas C., Dintrans P.V., Castillo C., Martinez Y. (2022). Factors associated with change in adherence to COVID-19 personal protection measures in the Metropolitan Region, Chile. PLoS One.

[15] Du Z., Wang L., Shan S., Lam D., Tsang T.K., Xiao J. (2022). Pandemic fatigue impedes mitigation of COVID-19 in Hong Kong. Proc Natl Acad Sci U S A.

[16] Petherick A., Goldszmidt R., Andrade E.B., Furst R., Hale T., Pott A. (2021). A worldwide assessment of changes in adherence to COVID-19 protective behaviours and hypothesized pandemic fatigue. Nat Hum Behav.

[17] Taylor S., Rachor G.S., Asmundson G.J.G. (2022). Who develops pandemic fatigue? Insights from latent class analysis. PLoS One.

[18] Msemburi W., Karlinsky A., Knutson V., Aleshin-Guendel S., Chatterji S., Wakefield J. (2023). The WHO estimates of excess mortality associated with the COVID-19 pandemic. Nature.

[19] Raisi-Estabragh Z., Cooper J., Salih A., Raman B., Lee A.M., Neubauer S. (2023). Cardiovascular disease and mortality sequelae of COVID-19 in the UK Biobank. Heart.

[20] Schöley J., Aburto J.M., Kashnitsky I., Kniffka M.S., Zhang L., Jaadla H. (2022). Life expectancy changes since COVID-19. Nat Hum Behav.

[21] (2020). Expert meeting on COVID-19 prevention in Japan. Opinion on COVID-19 Prevention.

[22] Furuse Y. (2021). Risk at mass-gathering events and the usefulness of complementary events during COVID-19 pandemic. J Infect.

[23] (2021). Harvard healthy buildings program. COVID-19 risk calculator - covid-19. https://covid-19.forhealth.org/covid-19-transmission-calculator/.

[24] Huang Jianxiang, Jones Phil (2021). COVID-19 infection risk calculator | HKU faculty of architecture. https://www.arch.hku.hk/research_project/covid-19-infection-risk-calculator/.

[25] Max Planck Institute for Chemistry (2020). https://www.mpic.de/4851094/risk-calculator.

[26] Hiroshima Prefectural Government (2022). Information on COVID-19: after the end of priority measures to prevent the spread of COVID-19, March 3, 2022. https://www.pref.hiroshima.lg.jp/site/2019-ncov/20220302.html.

[27] Hiroshima prefectural COVID-19 response headquarters, 60th meeting held on march 4, 2022 2022. https://www.pref.hiroshima.lg.jp/site/bousaisaigaijouhou/20220304.html.

[28] Ministry of Internal Affairs and Communications Statistics Bureau (2024). Population by Gender; Prefectures: Age (5-year age groups), Population by Gender.

[29] Sasanami M., Fujimoto M., Kayano T., Hayashi K., Nishiura H. (2023). Projecting the COVID-19 immune landscape in Japan in the presence of waning immunity and booster vaccination. J Theor Biol.

[30] Hellewell J., Russell T.W., Matthews R., Severn A., Adam S., Enfield L. (2021). Estimating the effectiveness of routine asymptomatic PCR testing at different frequencies for the detection of SARS-CoV-2 infections. BMC Med.

[31] Russell T.W., Townsley H., Abbott S., Hellewell J., Carr E.J., Chapman L.A.C. (2024). Combined analyses of within-host SARS-CoV-2 viral kinetics and information on past exposures to the virus in a human cohort identifies intrinsic differences of Omicron and Delta variants. PLoS Biol.

[32] Ministry of Internal Affairs and Communications (2023). Population, demographics, and household numbers based on the basic resident registery. https://www.soumu.go.jp/main_sosiki/jichi_gyousei/daityo/jinkou_jinkoudoutai-setaisuu.html.

[33] Okada Y., Ueda M., Nishiura H. (2024). Reconstructing the age-structured case count of COVID-19 from sentinel surveillance data in Japan: a modeling study. Int J Infect Dis.

[34] R Core Team (2023). R: the R project for statistical computing. https://www.r-project.org/.

[35] Gabry J., Češnovar R., Johnson A., Bronder S. (2023). R interface to CmdStan. https://mc-stan.org/cmdstanr/.

[36] Chang W., Cheng J., Allaire J.J., Sievert C., Schloerke B., Xie Y. (2024). Shiny: web application framework for R. https://shiny.posit.co/.

[37] Tupper P., Boury H., Yerlanov M., Colijn C. (2020). Event-specific interventions to minimize COVID-19 transmission. Proc Natl Acad Sci U S A.

[38] Adam D.C., Wu P., Wong J.Y., Lau E.H.Y., Tsang T.K., Cauchemez S. (2020). Clustering and superspreading potential of SARS-CoV-2 infections in Hong Kong. Nat Med.

[39] Furuse Y., Tsuchiya N., Miyahara R., Yasuda I., Sando E., Ko Y.K. (2022). COVID-19 case-clusters and transmission chains in the communities in Japan. J Infect.

[40] Oshitani H. (2020). The expert members of the national COVID-19 cluster taskforce at the Ministry of health LAWJ. Cluster-based approach to coronavirus disease 2019 (COVID-19) response in Japan, from february to april 2020. Jpn J Infect Dis.

[41] Lelieveld J., Helleis F., Borrmann S., Cheng Y., Drewnick F., Haug G. (2020). Model calculations of aerosol transmission and infection risk of covid-19 in indoor environments. Int J Environ Res Publ Health.

[42] Takayama Y., Shimakawa Y., Matsuyama R., Chowell G., Omori R., Nagamoto T. (2024). SARS-CoV-2 infection in school settings, Okinawa prefecture, Japan, 2021-2022. Emerg Infect Dis.

[43] Akaishi T., Kushimoto S., Katori Y., Sugawara N., Igarashi K., Fujita M. (2021). COVID-19 transmission at schools in Japan. Tohoku J Exp Med.

[44] Abaluck J., Kwong L.H., Styczynski A., Haque A., Kabir M.A., Bates-Jefferys E. (2022). Impact of community masking on COVID-19: a cluster-randomized trial in Bangladesh. Science.

[45] Elgersma I.H., Fretheim A., Elstrøm P., Aavitsland P. (2023). Association between face mask use and risk of SARS-CoV-2 infection: cross-sectional study. Epidemiol Infect.

[46] Gettings J., Czarnik M., Morris E., Haller E., Thompson-Paul A.M., Rasberry C. (2021). Mask use and ventilation improvements to reduce COVID-19 incidence in elementary schools - Georgia, November 16-December 11, 2020. MMWR Morb Mortal Wkly Rep.

[47] Sun K.S., Lau T.S.M., Yeoh E.K., Chung V.C.H., Leung Y.S., Yam C.H.K. (2022). Effectiveness of different types and levels of social distancing measures: a scoping review of global evidence from earlier stage of COVID-19 pandemic. BMJ Open.

[48] Andrasfay T., Wu Q., Lee H., Crimmins E.M. (2022). Adherence to social-distancing and personal hygiene behavior guidelines and risk of COVID-19 diagnosis: evidence from the Understanding America Study. Am J Publ Health.

[49] Buonanno G., Ricolfi L., Morawska L., Stabile L. (2022). Increasing ventilation reduces SARS-CoV-2 airborne transmission in schools: a retrospective cohort study in Italy's Marche region. Front Public Health.

