# Supplementary material for: Web-based real-time risk assessment of coronavirus disease 2019 infection in schools and social dining settings
Source: New Microbes New Infect. 2025 May 20;65:101600. doi: 10.1016/j.nmni.2025.101600 (PMC12159886; doi:10.1016/j.nmni.2025.101600)
Supplement: Multimedia component 1 [file mmc1.docx]

**Supplementary Materials.**

**Supplementary Methods:**

**Estimation of the time delay from date of infection to date of reporting**

We estimated the delay from infection to reporting in two steps, as in our previous study.[1] Briefly, in the first step, we estimated the delay from onset to reporting using epidemiological data from the Tokyo Metropolitan government. [2] These data contained individual-level information on dates of onset and dates of diagnosis. Let $D$ be part of this data set from Jan 20 to Feb 28, 2022. Assuming a parametric probability density function with parameter $\theta$ for the delay distribution from symptom onset, the discretized probability mass function as a function of time since the onset of illness can be described as:

$$p_{Onset\to Report}\left( t|\theta\right)={CDF}_{Onset\to Report}\left( t+1|\theta\right)-{CDF}_{Onset\to Report}\left( t|\theta\right),$$

and the likelihood function for observing $D$ can then be described as

$$L\left( \theta|D \right)=\prod_{i\in D} \frac{p_{Onset\to Report}\left( t_{i, report}-t_{i, onset}|\theta\right)}{p_{Onset\to Report}\left( T-t_{i, onset}|\theta\right)},$$

where $T$ is Feb 28, 2022. We assumed a Weibull-distributed delay function and estimated the best-fit estimate $\hat{\theta}$ using maximum likelihood estimation. The estimated shape and scale parameter for the Weibull distribution were as follows:

- Shape parameter = 2.224 (95%CI: 2.220–2.227)
- Scale parameter = 5.316 (95%CI: 5.313–5.320)

A parametric bootstrap was used to obtain the 95% confidence interval. For the analysis as described in the main text, the point estimates $\hat{\theta}$ were used.

In the second step, the lag distribution from dates of infection to dates of reporting was calculated as follows using $p_{Onset\to Report}\left( t|\hat{\theta} \right)$ and the reported incubation period of Omicron variants [3]:

$$g_{\tau}^{lag}=\sum_{s=0}^{\tau} p_{incubation}\left( s \right)p_{Onset\to Report}\left( \tau-s|\hat{\theta} \right).$$

**Calculation of the number of infected and susceptible people in school or social dining settings**

On the basis of $\phi_{l}$, which indicates the fraction of the population at location $l$ in each age group as shown in supplementary table 1 (below), we assumed that the age distribution of users at each location is proportional to the population size of age groups. This age distribution $\pi_{l}^{a}$ can be described as follows:

$$\pi_{l}^{a}=\frac{\phi_{l}^{a}N^{a}}{\sum_{a} \phi_{l}^{a}N^{a}},$$

where $N_{a}$ is the population size of age group $a$. Using $\pi_{l, a}$, the susceptible proportion of users at this location is:

$$s_{l, t}=\sum_{a} \pi_{l}^{a}\frac{S_{t}^{a}}{N^{a}},$$

where $S_{a}$ is the population susceptible to symptomatic COVID-19 infection in age group $a$ (estimated using the methodology of Sasanami et al. [4]) In the same manner, the number of infectious people in age group $a$ is:

$$J_{l, t}=\sum_{a} n_{l}\pi_{l}^{a}\sum_{\tau=0}^{\infty} g_{\tau}^{+}\circ\frac{I_{t-\tau}^{a}}{N^{a}},$$

where $n_{l}$ is the number of users of location $l$ (derived from Ueda et al.[4]), and $J_{l, t}$ is derived from the population-level infection counts and $g_{\tau}^{+}$, the probability of having infectivity after $\tau$ days since infection. Thus, the number of infections expected to occur at this location $l$, is

$$\mu_{l, t}=\beta_{l}\tau_{l}s_{l, t}J_{l, t}$$

where $\tau_{l}$ is the average duration of stay (in hours) at location $l$ (derived from Ueda et al. [5]). Under the assumption that

$$\beta_{l}=\beta_{L}, l\in L,$$

i.e., the per-hour transmission rates do not differ among locations in the same category $L$, then the total expected infections were calculated as:

$$\mu_{L, t}=\sum_{l\in L} \mu_{l, t}=\beta_{L}\sum_{l\in L} \tau_{l}s_{l, t}J_{l, t}.$$

We calculated $\mu_{L, t}$ for every day throughout the study period for use in the statistical inference of $\beta_{L}$.

**Priors and algorithm used in Bayesian inference by MCMC.**

Priors: We used weakly informative priors for all parameters estimated in our study as follows:

$$\beta_{L} \sim Half Cauchy\left( 0, 1 \right)$$

Algorithm: MCMC samples were obtained using Hamiltonian Monte Carlo algorithm with No-U-turn-sampler that is implemented in Stan software.

**Supplementary Information:**

**Description on “The Priority Measures to Prevent the spread of COVID-19”**

“The Priority Measures to Prevent the Spread of COVID-19 are regionally targeted, time-limited interventions aimed at suppressing transmission. They typically include requests or orders to:

- Shorten operating hours of restaurants, bars and other nightlife venues (often to around 8 pm).
- Prohibit or limit alcohol service in eateries and pubs.
- Cap attendance at concerts, sporting events and other gatherings (e.g. 50 % capacity or a fixed maximum number of people).
- Encourage teleworking and staggered commuting for businesses and public offices.
- Restrict use of meeting rooms, karaoke boxes, gyms, and other high-risk settings.
- Enhance testing and contact tracing in the designated area.
- Promote mask-wearing and hand hygiene through public campaigns.
- Provide financial support (subsidies) to affected businesses and individuals.

Relevant statement describing this measure by the Prime Minister of Japan as of June 17, 2021 can be found in the reference. [6]

**Epidemic situation in Hiroshima Prefecture from January to February 2022**

The data publicized by Hiroshima Prefectural Government shows that >97% cases that underwent variant screening were positive for Omicron BA.1 subvariant during this period. [7]

Another press release, also from the Hiroshima Prefectural Government, shows that the epidemic wave caused mainly by Omicron BA.1 subvariant starting in January reached the transient peak at 330-340 cases/week/100,000 population around Jan 25-30, 2022. This was followed by an overall decreasing trend, leading to the incidence rate to around 160-180 cases/week/100,000 population around Feb 24-28, 2022. [8]

**Supplementary Tables.**

**Supplementary Table 1: The fraction of the population in the age group of users of locations in which school or social dining settings occur.**

We defined the indicator vector $\phi_{l}=\left[ \phi_{l}^{0-9}, \phi_{l}^{10-19}, \ldots, \phi_{l}^{80+} \right]$ in the right column, which represents the fraction of the population at location $l$ in each age group. The definition of $\phi_{l}$ for subcategories within schools were based on the typical ages for attending each school level in Japan. For subcategories in social dining, we arbitrarily defined $\phi_{l}$ on the basis of the authors’ presumptions. The classification of settings followed that of Ueda et al. [5]

| **Schools** | $\phi_{l}^{a}=\left[ \phi_{l}^{0-9}, \phi_{l}^{10-19}, \ldots, \phi_{l}^{80+} \right]$ |
| --- | --- |
| Kindergarten | $\left[ 0.6, 0.0, 0.0, 0.0, 0.0, 0.0, 0.0, 0.0, 0.0 \right]$ |
| Elementary school | $\left[ 0.4, 0.3, 0.0, 0.0, 0.0, 0.0, 0.0, 0.0, 0.0 \right]$ |
| Middle school | $\left[ 0.0, 0.3, 0.0, 0.0, 0.0, 0.0, 0.0, 0.0, 0.0 \right]$ |
| High school | $\left[ 0.0, 0.3, 0.0, 0.0, 0.0, 0.0, 0.0, 0.0, 0.0 \right]$ |
| University | $\left[ 0.0, 0.1, 0.3, 0.0, 0.0, 0.0, 0.0, 0.0, 0.0 \right]$ |
| Cram school | $\left[ 0.2, 0.4, 0.4, 0.0, 0.0, 0.0, 0.0, 0.0, 0.0 \right]$ |
| **Social dining** |  |
| Restaurant | $\left[ 0.0, 1.0, 1.0, 1.0, 1.0, 1.0, 1.0, 1.0, 0.0 \right]$ |
| Alcohol serving eating and drinking establishment | $\left[ 0.0, 0.0, 1.0, 1.0, 1.0, 1.0, 1.0, 0.0, 0.0 \right]$ |
| Serviced entertaining bars and escort clubs | $\left[ 0.0, 0.0, 1.0, 1.0, 1.0, 1.0, 1.0, 0.0, 0.0 \right]$ |

**References:**

[1] Okada Y, Nishiura H. Estimating the effective reproduction number of COVID-19 from population-wide wastewater data: An application in Kagawa, Japan. Infect Dis Model 2024;9:645–56.

[2] Tokyo Metropolitan Government COVID-19 Information Website. Webpage in Japanese n.d. https://stopcovid19.metro.tokyo.lg.jp/.

[3] National Institute of Infectious Diseases. Estimation of Incubation Period for SARS-CoV-2 Mutant B.1.1.529 Strain (Omicron Strain): Preliminary Report 2022. https://www.niid.go.jp/niid/ja/2019-ncov/2551-cepr/10903-b11529-period.html.

[4] Sasanami M, Fujimoto M, Kayano T, Hayashi K, Nishiura H. Projecting the COVID-19 immune landscape in Japan in the presence of waning immunity and booster vaccination. J Theor Biol 2023;559:111384.

[5] Ueda M, Hayashi K, Nishiura H. Identifying High-Risk Events for COVID-19 Transmission: Estimating the Risk of Clustering Using Nationwide Data. Viruses 2023;15:456.

[6] Prime Minister of Japan and His Cabinet. [COVID-19] Press Conference by the Prime Minister regarding the Novel Coronavirus (Speeches and Statements by the Prime Minister) 2021. https://japan.kantei.go.jp/99_suga/statement/202106/_00008.html (accessed April 25, 2025).

[7] Hiroshima Prefectural Government: Information on the SARS-CoV-2 variants in Hiroshima Prefecture 2025. https://www.pref.hiroshima.lg.jp/site/hcdc/henikabu.html (accessed April 25, 2025).

[8] Hiroshima Prefectural Government. Information on COVID-19: After the end of priority measures to prevent the spread of COVID-19, March 3, 2022 (in Japanese) 2022. https://www.pref.hiroshima.lg.jp/site/2019-ncov/20220302.html.
